# Trends in the use and costs of round-the-clock long-term care in the last two years of life among old people between 2002 and 2013 in Finland

**DOI:** 10.1186/s12913-017-2615-3

**Published:** 2017-09-19

**Authors:** Leena Forma, Marja Jylhä, Jutta Pulkki, Mari Aaltonen, Jani Raitanen, Pekka Rissanen

**Affiliations:** 1 Faculty of Social Sciences (health sciences) and Gerontology Research Center (GEREC), University of Tampere, 33014 Tampere, Finland; 2 Institute for Advanced Social Research, University of Tampere, Tampere, Finland; 3 UKK-Institute for Health Promotion, Tampere, Finland

**Keywords:** Long-term care, Use and costs, Last years of life, Time trend

## Abstract

**Background:**

The structure of long-term care (LTC) for old people has changed: care has been shifted from institutions to the community, and death is being postponed to increasingly old age. The aim of the study was to analyze how the use and costs of LTC in the last two years of life among old people changed between 2002 and 2013.

**Methods:**

Data were derived from national registers. The study population contains all those who died at the age of 70 years or older in 2002–2013 in Finland (*N* = 427,078). The costs were calculated using national unit cost information. Binary logistic regression and Cox proportional hazard models were used to study the association of year of death with use and costs of LTC.

**Results:**

The proportion of those who used LTC and the sum of days in LTC in the last two years of life increased between 2002 and 2013. The mean number of days in institutional LTC decreased, while that for sheltered housing increased. The costs of LTC per user decreased.

**Conclusions:**

Use of LTC in the last two years of life increased, which was explained by the postponement of death to increasingly old age. Costs of LTC decreased as sheltered housing replaced institutional LTC. However, an accurate comparison of costs of different types of LTC is difficult, and the societal costs of sheltered housing are not well known.

## Background

The use of long-term care (LTC) among old people is concentrated into the last years and months of life, and the use of LTC at the end of life is higher in older old than younger old age [1–4]. Deaths are increasingly postponed to later old age: in Finland in 1994 ca. 32,500 deaths occurred at the age of 70+ and 4100 (12.6%) at the age of 90+, while in 2014 the corresponding figures were 38,800 and 9500 (24.5%) respectively (Statistics Finland 2016). Thus the last years of life are being lived at an older age than before, and consequently the use of LTC near the end of life will probably grow [5].

A number of political programs in Finland have attempted to change highly institutionalized LTC practices and have recommended community-based services instead [6, 7], as has also happened in many other countries [8, 9]. In practice, the proportion of old people using LTC has remained close to 10% since the 1990s, but the use of institutional care has decreased, and that of sheltered housing (service housing, classified as non-institutional care) has increased in Finland [10–12]. Earlier studies on trends in LTC use have focused on old people in general, but trends in LTC use among those who use the services most, old people at the end of life, are not known. As the number of such people is increasing in many countries, knowing the trends is important for understanding the distribution of service use and for planning how to respond to increasing needs in the near future.

There are three types of round-the-clock LTC in Finland: inpatient care in health center wards (primary care hospitals, which also provide short-term care), residential homes, and sheltered housing with 24-h assistance (referred to hereafter as sheltered housing). Of these, health centers and residential homes are institutional settings, and sheltered housing provides housing and closely related services. In the past, these LTC facilities were presented as forming a hierarchy: people with the smallest care needs were predominantly cared for in sheltered housing, and those with the greatest needs in health centers [13]. However, this hierarchy has been remodeled, and nowadays residential homes and sheltered housing generally respond to similar needs. Those with the highest needs or in need of medical care are cared for in health centers. However, the client profiles in these services overlap to some extent.

Municipalities are responsible for providing LTC for their citizens in Finland [14]. The largest proportion of costs is paid for by municipal taxes (84% for health centers and 72% for residential homes), and the users of services pay the rest out of their own pockets [15]. National information on the funding of sheltered housing is not available, but in these settings residents pay for a variety of day-to-day commodities, e.g. medicines, out of their own pockets. Since they are entitled to apply for reimbursements of medical and housing expenses from the Social Insurance Institution (SII), responsibility for the funding of LTC is being shifted from local- to national-level welfare systems, and to service users themselves [16]. The unit costs of health centers are highest and those of sheltered housing lowest (Table 1), but these costs do not include all the same cost items, and therefore the differences in cost are not clear at the societal level. From the viewpoint of municipalities, sheltered housing has been found to be 22% cheaper than institutional care, but when SII reimbursement is taken into account, the total costs of sheltered housing are 9% cheaper than those of institutional care [17]. Still, there is a lack of information about costs paid by clients, and consequently about total societal costs.

**Table 1:** Unit costs of different types of LTC

	Cost items	€ 2011^b^	€ 2013^c^	Total costs in € 2013
Health center	Staff, administration, meals, clothes, care supplies, housing, medicines,^a^ TVs, hygiene products, phones	257	271	271
Residential home	Staff, administration, meals, clothes, care supplies, housing, medicines,^a^ TVs, hygiene products, phones	185	195	195
Sheltered housing	Staff, meals, care supplies^a^	131	138	156
+ housing costs			13.60^d^	
+ medicine costs			4.00^e^	

^a^[21] The unit costs for the care of old people reported by Kapiainen et al. [20] are mainly based on this report

^b^Costs per day in LTC provided by municipality [20]

^c^Costs were converted to their 2013 equivalent values according to the price index of public expenditure for health and social services (Statistics Finland)

^d^[22]

^e^COCTEL data: medicine costs per day in the last two years of life among those who died in 2013 and were community-dwelling for at least one day

We describe and analyze the use and societal costs of round-the-clock LTC among old people during the last two years of life, and how those uses and costs changed between 2002 and 2013. These were analyzed for long-term care in total, and separately for health center inpatient wards, residential homes, and sheltered housing. During the study period the structure of LTC continued to change so that care was shifted from institutions to the community, and death was being postponed to increasingly old age. These changes are current in many countries, and the results of this study add knowledge about LTC use in a group with the highest care needs. This study was conducted as part of the project entitled “New Dynamics of Longevity and the Changing Needs for Services” (COCTEL).

## Methods

### Study population

The study population was drawn from the Causes of Death Register (Statistics Finland). It consists of all those who died at the age of 70 years or over in 2002–2013 in Finland. The cutoff age of 70 was chosen because both the risk of death (Statistics Finland) and healthcare expenditures per resident start to increase at around 70 years of age in Finland [18]. Use of LTC was examined for the last 730 days of life. Thus the data include decedents for 12 years and service use for 14 years since 2000.

### Data sources

The data on LTC use were derived from the Care Register for Healthcare and Care Register for Social Welfare (National Institute for Health and Welfare). The information from these registers was linked using unique Personal Identification Codes. A more detailed description of the data collection has been given elsewhere [19]. Days in care were calculated for each individual on the basis of dates of admission to and discharge from care.

Permission to access and use the register data was obtained from both register authorities. The research plan was approved by the Pirkanmaa Hospital District Ethics Committee.

### Measures

Use and costs of round-the-clock LTC were analyzed in total and separately for three types of LTC: (1) health center inpatient wards, if the person had a continuous length of stay of 90 days or over; (2) residential homes; (3) sheltered housing with 24-h assistance. The LTC in total is the sum of these three types of LTC.

Three outcome variables were created for LTC in total and for each type of LTC: (1) any use, where 1 = used at least once in the last 730 days of life, and 0 = did not use in the last 730 days of life; (2) days in care (for health centers days vary from 90 to 730 and for other service types from 1 to 730); (3) costs of care in the last 730 days of life.

We multiplied the number of days in different types of LTC by their daily unit costs, derived from a national report [20] (Table 1). The unit costs for residential homes and health centers are gross costs caused by the use of services. We used the unit costs for the year 2013 for all years. Costs were converted from year 2011 values to their 2013 equivalent values according to the price index of public expenditure for health and social services (Statistics Finland).

The unit cost of sheltered housing included staff, meals, and materials, but excluded housing, medicines, and some purchased services [21]. Instead, these were included in the unit costs of health centers and residential homes (cost items are described in Table 1). We estimated the cost of housing (an average of €13.60 per day in sheltered housing) using statistics from the SII [22]. The mean cost of prescribed outpatient medicines was derived from the SII’s individual-level register data. This was €4 per day among those who died in 2013 and who were community-dwelling^1^ for at least one day in the last two years of life. We added these cost items to the unit cost of sheltered housing to make it more comparable with other types of LTC and to represent the societal costs, like the costs of other types of LTC do. Nonetheless, many other costs paid by users of sheltered housing were excluded, such as for clothing, TVs, hygiene products, and phones. In residential homes and health centers these costs are covered (see Table 1). The unit cost for sheltered housing is an underestimation, but it is the best estimation available, and this limitation must be kept in mind when interpreting the results. The unit costs of sheltered housing are difficult to estimate, since there is variation in the content of service packages offered to clients.

### Analyses

Analyses were performed for the whole study population and by age group (70–79, 80–89 and 90+ years). Chi-square tests were used to test the differences in the proportions of service users and genders between the years of death. One-way ANOVA was used to assess the change in the mean age at death during the study period. Independent samples median tests were used to test the differences in the number of days in care, and in the costs between those who died in different years. The number of days in and costs of LTC were analyzed among the users of services. In addition, the sum of days in LTC is presented. Binary logistic regression analyses were performed to assess the probability of using LTC by year of death. Age and gender were adjusted for.

The costs of LTC in our data include a lot of zeroes, and the distributions are U-shaped, meaning that there were many individuals with few or no days in care (for health centers 0 or ≥90) and many who were in care for 730 or almost 730 days. Survival methods such as Cox proportion hazard models may be employed when the data are skewed or multimodal, have heavy tails, or consist of excess zeroes [23]. These models have been used even when the censoring does not have to be corrected [24]. In comparisons of different regression models for analyzing the cost data, proportional hazard models have been shown to perform well [25] when the proportional hazards assumption is met [24].The proportional hazards assumption means that the survival curves for two different levels of a covariate are proportional over time (i.e. constant relative hazard) [26].

We employed Cox proportional hazard models to analyze the development of the costs of LTC per user between 2002 and 2013. Costs were considered a “survival time” variable, and those who did not have “survival time”, i.e. whose costs were 0, were dropped from the models. None of the observations was treated as censored, as for all the end point was death. Age, gender, and year of death were independent variables. In addition, models including an interaction term (age * year of death) were run to find out whether the effect of age on costs differed between the years. The negative coefficient estimate (hazard ratio (HR) <1.00) for the proportional hazard model indicates a decreased hazard of reaching total costs, hence an increase in total cost [25]. Therefore, we present the inverses of HRs to make the interpretation easier, i.e. a higher value means higher costs.

## Results

### Descriptives

**Fig. 1:**
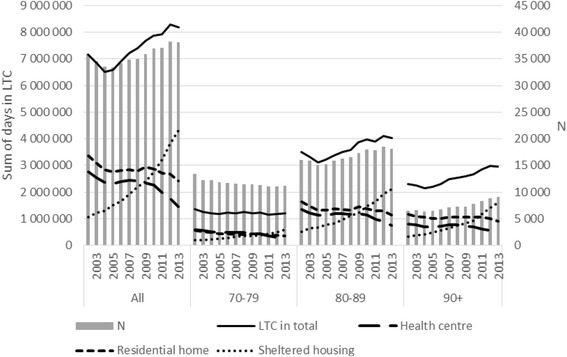
The sum of days in LTC in the last two years of life and the number of decedents by age group from 2002 to 2013: *N* = 427,078

The data included 427,078 persons. The mean age at death increased from 82.3 to 83.8 years between 2002 and 2013 (*p* < .001), and the proportion of women decreased from 59.1% to 56.5% (*p* < .001). The number of people who died at the age of 70–79 years decreased and that of older people increased during the study period (Fig. 1).

### The proportion of LTC users

**Fig. 2:**
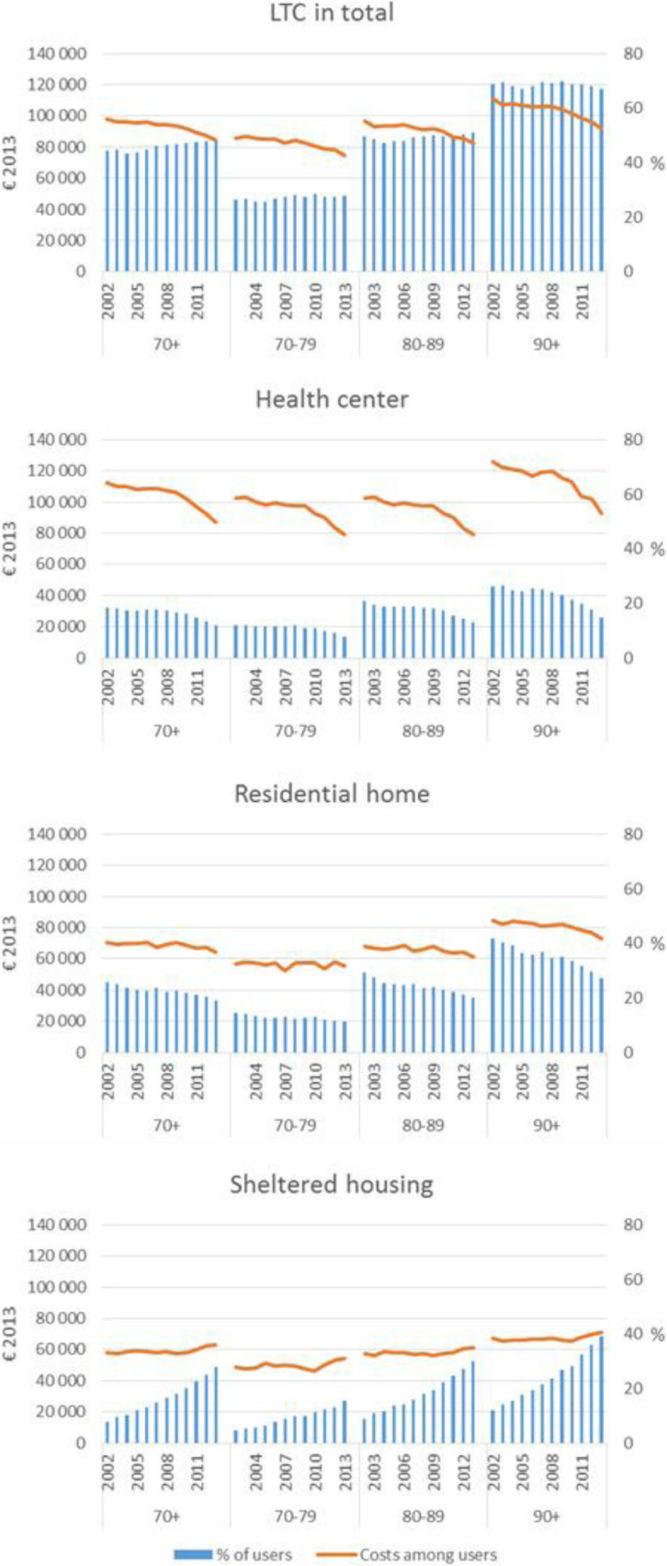
The proportion of LTC users out of all decedents, and the mean costs of LTC (€ 2013) among users by age group from 2002 to 2013 (the costs of health centers are higher than those of LTC in total, since the number of days in a health center varies from 90 to 730 and for other types of LTC from 1 to 730)

The proportion of those who used LTC in total increased (*p* < .001) over the study period among those who died at the age of 70+. It increased in younger age groups, but decreased (*p* < .001) among the oldest (90+) (Fig. 2). The use of institutional care (health centers and residential homes) decreased (*p* < .001), while the use of sheltered housing increased (*p* < .001) in all age groups.

The likelihood of using LTC in total remained approximately at the same level between 2002 and 2013 when age was adjusted for (stepwise analyses not shown) (Table 2). The decrease in the use of institutional care and the increase in use of sheltered housing were also shown in these analyses. Use of all types of LTC was more common among older than younger decedents and among women than men (Table 2).

**Table 2:** The association of age, gender, and year of death with any use of LTC, logistic regression analyses: *N* = 427,078

	LTC	Health center	Residential home	Sheltered housing
	OR (95% CIs)	OR (95% CIs)	OR (95% CIs)	OR (95% CIs)
Age	1.10 *** (1.10, 1.10)	1.04 *** (1.04, 1.04)	1.07 *** (1.07, 1.07)	1.06 *** (1.06, 1.06)
Gender (ref. man)	1.54 *** (1.52, 1.56)	1.48 *** (1.45, 1.51)	1.23 *** (1.21, 1.25)	1.29 *** (1.27, 1.31)
Year of death				
2002 (ref.)				
2003	0.98 (0.95, 1.01)	0.97 (0.93, 1.00)	0.93 *** (0.90, 0.97)	1.22 *** (1.16, 1.29)
2004	0.94 *** (0.91, 0.97)	0.92 *** (0.89, 0.96)	0.88 *** (0.85, 0.91)	1.33 *** (1.27, 1.41)
2005	0.95 *** (0.92, 0.98)	0.92 *** (0.88, 0.96)	0.83 *** (0.80, 0.86)	1.58 *** (1.50, 1.66)
2006	0.98 (0.95, 1.01)	0.93 *** (0.90, 0.97)	0.81 *** (0.79, 0.84)	1.73 *** (1.64, 1.82)
2007	1.02 (0.99, 1.05)	0.92 *** (0.89, 0.96)	0.84 *** (0.81, 0.87)	1.98 *** (1.89, 2.08)
2008	1.03 (1.00, 1.06)	0.91 *** (0.87, 0.95)	0.77 *** (0.75, 0.80)	2.27 *** (2.17, 2.38)
2009	1.04 ** (1.01, 1.08)	0.86 *** (0.83, 0.90)	0.78 *** (0.76, 0.81)	2.51 *** (2.40, 2.64)
2010	1.03 * (1.00, 1.07)	0.82 *** (0.79, 0.85)	0.75 *** (0.72, 0.78)	2.88 *** (2.75, 3.01)
2011	1.04 (1.00, 1.07)	0.74 *** (0.71, 0.77)	0.71 *** (0.68, 0.73)	3.33 *** (3.18, 3.49)
2012	1.03 * (1.00, 1.06)	0.66 *** (0.63, 0.69)	0.66 *** (0.64, 0.68)	3.77 *** (3.60, 3.95)
2013	1.04 (1.00, 1.07)	0.57 *** (0.54, 0.59)	0.61 *** (0.59, 0.63)	4.38 *** (4.19, 4.58)
Nagelkerke R^2^	0.151	0.039	0.069	0.091

*p < .05, **p < .01, ***p < .001

*LTC* long-term care, *OR* odds ratio, *CI* confidence interval

### The number of days in, and the costs of, long-term care

The number of deaths at the age of 70+ decreased from 2002 to 2005, and increased remarkably from 2006 to 2013. Therefore the sum of days in LTC in total first decreased and then increased (Fig. 1). The sum of days in LTC decreased among the youngest (70–79) but increased in the older age groups.

Among those who used LTC, the number of days in LTC in total increased slightly (mean 450 in 2002 and 448 in 2013, median 540 and 549 respectively, *p* < .001) in the study period, but the costs per user decreased (*p* < .001) (Fig. 2). The number of days, as well as the costs of care in health centers and residential homes, decreased (*p* < .001) from 2002 to 2013. Conversely, the number of days in and costs of sheltered housing increased (*p* < .001).

We ran Cox proportional hazard models to find out how the costs of LTC developed during the study period among service users. The costs of LTC in total decreased systematically from 2002 to 2013 (Table 3). The costs of health centers decreased, and the costs of sheltered housing increased. The costs of residential homes did not change during the study period. Older people and women had a higher probability of higher costs for each type of LTC than younger people and men (Table 3).

**Table 3:** The association of age, gender, and year of death with costs of LTC among users, Cox proportion hazard analyses

	LTC in total	Health center	Residential home	Sheltered housing
N	196,461	69,958	96,722	71,404
	iHR (95% CIs)	iHR (95% CIs)	iHR (95% CIs)	iHR (95% CIs)
Age	1.01 *** (1.01, 1.01)	1.01 *** (1.01, 1.01)	1.02 *** (1.02, 1.02)	1.02 *** (1.02, 1.02)
Man (ref. woman)	1.26 *** (1.25, 1.27)	1.17 *** (1.15, 1.19)	1.29 *** (1.27, 1.31)	1.25 *** (1.23, 1.27)
Year of death (ref. 2002)				
2003	0.97 ** (0.95, 0.99)	0.97 (0.94, 1.01)	0.99 (0.96, 1.02)	0.99 (0.94, 1.04)
2004	0.97 ** (0.95, 0.99)	0.98 (0.94, 1.01)	1.00 (0.97, 1.03)	1.04 (0.99, 1.09)
2005	0.95 *** (0.93, 0.98)	0.96 * (0.92, 0.99)	1.00 (0.97, 1.03)	1.04 (0.99, 1.09)
2006	0.96 *** (0.94, 0.98)	0.95 ** (0.92, 0.99)	1.02 (0.99, 1.05)	1.05 (1.00, 1.10)
2007	0.93 *** (0.91, 0.95)	0.94 *** (0.91, 0.98)	0.99 (0.96, 1.02)	1.04 (0.99, 1.09)
2008	0.93 *** (0.91, 0.95)	0.94 *** (0.91, 0.97)	1.01 (0.98, 1.05)	1.06 ** (1.02, 1.11)
2009	0.91 *** (0.89, 0.93)	0.93 *** (0.90, 0.96)	1.05 ** (1.02, 1.08)	1.06 * (1.01, 1.11)
2010	0.87 *** (0.85, 0.89)	0.87 *** (0.84, 0.90)	1.02 (0.99, 1.05)	1.07 *** (1.03, 1.12)
2011	0.82 *** (0.81, 0.84)	0.83 *** (0.80, 0.86)	1.02 (0.99, 1.05)	1.12 *** (1.07, 1.17)
2012	0.79 *** (0.77, 0.81)	0.77 *** (0.75, 0.80)	1.04 ** (1.01, 1.08)	1.20 *** (1.15, 1.26)
2013	0.73 *** (0.71, 0.74)	0.71 *** (0.68, 0.74)	0.99 (0.96, 1.02)	1.28 *** (1.23, 1.33)

*p < .05, **p < .01, ***p < .001

*LTC* long-term care, *iHR* inverse of hazard ratio, *CI* confidence interval

We also ran Cox proportional hazard models including the interaction term (age * year of death) to find out whether the effect of age on LTC costs changed during the study period (analyses not shown). The effect of this interaction term was statistically significantly associated with the costs of LTC in total for the years 2010–2013 (for all years inverse of HR 0.996, 95% confidence intervals (CIs) 0.993, 0.999), and with the costs of residential homes in 2012 and 2013 (for both years inverse of HR 0.993, 95% CIs 0.989, 0.998). This indicates that in these years the effect of age on LTC costs was 0.4% and on residential home costs 0.7% weaker than in 2002.

## Discussion

The aim of this study was to describe and analyze how the use and costs of round-the-clock LTC among old people in the last two years of life changed between 2002 and 2013. We found that the proportion of LTC users increased among those who died at the age of 70 years or over, but the increase was not clear when age was adjusted for. This implies that the increase was due to the change in the age structure of old people, i.e. the postponement of death to older ages. Use and costs of LTC are higher among older old than younger old people, and the effect of age on use and costs of LTC did not change much during the study period. As the last years of life are being lived at a greater age than before, functional and cognitive disability is probably greater than previously [27], and the period during which care is needed may be longer [28].

The composition of LTC changed during the study period. The use of institutional LTC (health centers and residential homes) decreased, and the use of sheltered housing increased. The number of days in LTC in total among users did not change much, but the costs of LTC in total per user decreased. This was mainly due to the replacement of institutional LTC with sheltered housing, the costs of which are lower than those of institutional LTC. However, the unit costs of sheltered housing are difficult to estimate, as the costs and contents of services vary between sheltered housing units. In particular, the costs that clients pay out of their own pockets are not well known, and not all of them are included in the unit costs used here. We used the national unit costs, but since these do not include the costs of medicines and housing, we estimated their impact on costs. Consequently, the difference between the daily costs of residential homes and sheltered housing diminished from €57 to €39. However, the unit cost of sheltered housing is probably an underestimation.

The costs of LTC naturally depend heavily on the unit costs used. We used the same unit costs (2013) for all years, although costs may have changed during the study period. Furthermore, if there were significant changes in the content of care, or in the need for care among users, this may also have led costs to change. The unit costs used here are the national average costs, while the actual costs vary somewhat between municipalities and service providers [29].

The role of sheltered housing clearly increased during the study period in the care of old people in their last two years of life, as in the care of old people in general [10–12, 30]. This shift is mainly due to changes in the supply of services, rather than to the preferences of old people. If an individual in sheltered housing needs as much care and as many services as someone in a residential home, the costs of their care are likely to increase, becoming near or equal to the costs of care in a residential home [16]. Earlier studies have reported remarkable differences in care needs between residents in different types of LTC [30], but in our data people using different types of LTC did not differ much in terms of age, gender, or dementia diagnosis (data not shown).

According to our previous analyses, old people are not living until their deaths in sheltered housing as often as in residential homes. Also, the number of transitions to different care facilities (commonly hospitals) in the last year of life has been higher from sheltered housing than from residential homes [31, 32]. In addition to possible problems in the continuity of care, multiple admissions to hospital care also increase end-of-life care costs. We also analyzed the variation in the total costs of social and health services between those living in residential homes and those in sheltered housing, and no difference was found [33].

The use of registers, which are considered reliable [34, 35], is a strength of this study. In analyzing the costs of LTC we employed Cox proportional hazard models, which are not a very established way to make such an analysis. However, according to previous econometric comparisons, it is a suitable method for the analysis of complex cost distributions, although an essential requirement is that the proportional hazards assumption be met [23, 25]. We examined this assumption by considering the “survival curves” of costs between years of death, and found that they were proportional (parallel) at all points of the cost distribution. In addition, Cox regression has been found to be valid for analyzing costs when the data are not censored [36]; this was the case in our study, where the follow-up was two years for all.

The focus of this study was on round-the-clock LTC; thus home care and informal care, which are important parts of LTC, were not included. The proportion of home care users in the last two years of life increased from 19% in 2002 to 21% in 2008 in Finland [37]. Although there is a policy emphasis on living at home for as long as possible, the stability of the number of days in LTC among the users in this study suggests that old people may not be able to live longer in their own homes with the current level of home care.

Individual characteristics, other than age and gender, were not controlled for in our analyses, although disease and disability [38, 39], living arrangements and socio-economic status [40, 41], and availability of informal care [42, 43] are known to be important determinants of the use and costs of LTC. In this study, however, the focus was on time trends in the use and costs of LTC, not on individual determinants.

## Conclusions

A remarkable change in the arrangement of round-the-clock LTC for old people in the last two years of life took place in Finland between 2002 and 2013. The number of days in LTC increased slightly, but the costs of LTC in total per user decreased during the study period. There was a shift from institutional care to sheltered housing. However, reliably comparing the costs of different types of LTC is difficult, because they do not include the same cost items. We estimated the societal costs of sheltered housing, but still could not include them all. In particular, the costs paid by the service users are difficult to estimate. The increased use of sheltered housing raises concerns about shifting the financial responsibility onto clients. Use of LTC in total increased, as the age structure of the decedents changed. As death continues to be postponed to older ages, the need for, and the use and costs of, LTC will probably increase. Choices should be made about how to supply good care for old people in ways that will share the costs of LTC equitably.

## Abbreviations

CI: Confidence interval
COCTEL: New Dynamics of Longevity and the Changing Needs for Services project
HR: Hazard ratio
iHR: inverse of hazard ratio
LTC: Long-term care
OR: Odds ratio
SII: Social Insurance Institution of Finland

## Footnotes

1 People were considered community-dwelling if they lived in a private home or sheltered housing, which is classified as non-institutional care.
